# Survival and hospitalization among patients with acute myeloid leukemia treated with azacitidine or decitabine in a large managed care population: a real-world, retrospective, claims-based, comparative analysis

**DOI:** 10.1186/2162-3619-3-10

**Published:** 2014-03-25

**Authors:** B Douglas Smith, Charles L Beach, Dalia Mahmoud, Laura Weber, Henry J Henk

**Affiliations:** 1 Sidney Kimmel Comprehensive Cancer Center at Johns Hopkins, Baltimore, MD, USA; 2 Hematology/Oncology Clinical Research and Development, Celgene, Summit, NJ, USA; 3 Global Pricing and Market Access, Celgene, Summit, NJ, USA; 4 Health Economic and Outcomes Research, Optum, Eden Prairie, MN, USA

**Keywords:** Acute myeloid leukemia, Survival, Hospitalization, Azacitidine, Decitabine, Managed care, Real-world

## Abstract

**Background:**

This study examined patient outcomes using real world data for acute myeloid leukemia (AML) patients initiating treatment.

**Methods:**

A retrospective, administrative claims-based, comparative analysis was developed to study outcomes for AML patients initiating treatment with decitabine or azacitidine between January 2006 and June 2012.

**Results:**

Treatment with azacitidine was associated with a longer median overall survival (10.1 versus 6.9 mos., p = 0.007) and a lower risk of hospitalization (HR 0.787, p = 0.02) compared to treatment with decitabine.

**Conclusions:**

This analysis of the outcomes of real-world treatment of AML patients with demethylating agents suggests that azacitidine may result in clinically superior outcomes than decitabine.

## Background

Acute myeloid leukemia (AML) is the most common form of leukemia in US adults, is associated with the largest number of deaths [1], and is particularly challenging for clinicians treating elderly patients who often have limited treatment options due to their age and co-morbid conditions. The National Comprehensive Cancer Network (NCCN) guidelines consider patients aged 60 years or older eligible for high-intensity induction therapy in limited circumstances [1]. This is in part due to the finding that the percentage of patients who demonstrate clinical characteristics making them fit for high-intensity chemotherapy decreases with age [2]. In fact, the use of traditional, standard-dose induction chemotherapy for the treatment of AML has been shown to decrease with age [3,4]. In US cancer trials, it is common that older patients are excluded from participating based on their age at diagnosis [5].

The limitations noted are not without reason as intensive, chemotherapy-based induction carries a comparably higher risk of death in older patients compared with younger ones (overall survival [OS]; ranges from 3.5 months [>75 years of age] to 18.8 months [≥56 to ≤75 years of age]; overall mortality hazard ratio [HR] of 1.2-1.3) [6,7]. Yet, these concerns must be weighed carefully against the finding that even in those patients who elect intensive-treatment there is a limited overall survival benefit. With a median age at diagnosis of AML in the US of 66 years [6], the challenge faced by clinicians in the treatment of elderly patients with newly diagnosed AML is quite real.

Hypomethylating agents have been used as lower-intensity AML induction treatments, particularly in patients age ≥60 years [1]. However, outcome data from clinical studies are inconsistent regarding the use of both azacitidine (Vidaza®, Celgene Corporation, Summit, NJ) and decitabine (Dacogen®, Eisai Inc., Woodcliff Lake, NJ) in the treatment of the elderly population with AML. A prospective trial examining the use of azacitidine (AZA) to treat elderly patients with AML found an increased OS when compared to conventional or best care (median OS 24.5 mos., 15.0 mos., respectively; HR 0.58; 95% CI 0 · 43—0 · 77; p = 0.0001) [8]. Additionally, a post-hoc analysis of a subset of elderly patients with AML (refractory anemia with excess blasts in transformation [RAEB-T] patients) found a longer overall survival for AZA-treated patients compared to conventional care (OS 24.5 mos., 16 mos. respectively; HR 0.47; 95% CI, 0.28 to 0.79; p = 0.005) [9]. A recent retrospective, single-site study in elderly patients also found significant survival advantage in favor of azacitidine vs. best supportive care although no difference in the improvement of OS with azacitidine when compared with intensive chemotherapy despite the finding that azacitidine was associated with less hospitalization (median of 0.5 days versus 56 days, respectively in the first 3 months) and fewer RBC and platelet transfusions (median of 2.7 vs. 7 per month respectively during the first 3 mos.) [10]. Likewise, the use of decitabine in elderly patients with AML showed a clinical benefit in OS (albeit non- significant) compared to supportive care alone (median OS 7.7 vs. 5.0 months respectively, HR 0.85, p = 0.108) [11]. In addition, two, open-label, single arm studies found a similar median OS rates with decitabine (DAC) therapy in elderly AML patients (median OS of 7.7 and 5.5 months) [12,13]. The inconsistent results are further difficult to interpret in that the AML populations examined were different (approximately 50-75% of AZA-treated patients had had <30% blasts whereas only 3.1-28% DAC-treated patients met this criterion) [8,9,11-13].

Retrospective comparisons of hypomethylating agents and chemotherapy in elderly patients often show higher response rates with chemotherapy-based regimens, but not an increase in survival [14]. These outcomes are conflicted with the finding that hypomethylating agents result in improved OS in controlled trials and raise the concern of loss of efficacy when using this class of agents outside of clinical trials. In fact, the potential importance of hypomethylating agents for the treatment of elderly patients with AML may be not fully realized. Understanding real-world results may better frame the treatment approaches for this group of patients. The purpose of this study is to examine the real world translation of the use of hypomethylating agents to treat elderly patients with AML by assessing patient outcomes for those initiating treatment with azacitidine or decitabine. The objectives were to describe overall survival for patients who initiated each treatment, assess the clinical impact of the treatment including describing the time to hospitalization after treatment initiation, and the occurrence of infections and bleeding events.

## Results

### Demographic and clinical characteristics

Of the 1,922 commercial or Medicare Advantage enrollees who initiated treatment for AML, a total of 487 patients (AZA, n = 288, DEC, n = 199) were determined eligible for analysis. Of the patients excluded, 513 met the criteria of not being continuously enrolled 6 months prior to index and 922 did not have a claim for AML diagnosis 6 months prior to or within 60 days of index date.

Demographic and clinical characteristics for each cohort are in Table 1. The mean age of the cohorts (AZA 70.3 ± 11.8 years, DEC 69.4 ± 11.6 years) indicated the populations were elderly with over 70% of the each cohort being ≥65 years of age. Most patient characteristics were similar between cohorts. There were two notable differences between the groups: the decitabine cohort had significantly more hospitalizations (62% AZA, 71% DEC; p = 0.0323) and a higher baseline mean Charlson comorbidity score (3.0 AZA, 3.4 DEC; p = 0.0140) noted during the pre-index period.

**Table 1:** Demographic and clinical characteristics

	**Azacitidine (n = 288)**	**Decitabine (n = 199)**	**p-value**
**Demographic characteristics**			
Age, mean (SD)	70.3 years (11.8)	69.4 years (11.6)	0.3890
18-64 years old	25.3	28.6	--
65+ years old	74.7	71.4	--
Gender (% male)	59.0	54.8	0.3510
Insurance Type			0.2450
Commercial Insurance (%)	46.5	41.2	--
Medicare Advantage (%)	53.5	58.8	--
**Clinical characteristics**			
MDS diagnosis *, %	54.5	51.9	0.549
RBC transfusion*, %	51.0	54.8	0.4185
ESA utilization**	26.4	25.1	0.754
G/GM-CSF utilization**	18.1	19.1	0.771
Charlson Comorbidity Index* mean (SD)	3.0 (1.7)	3.4 (1.8)	0.0140
Hospitalization*	61.5	70.9	0.0323

*baseline period, 6 months pre-index.

**follow-up period, post-index.

### Overall survival

**Figure 1:**
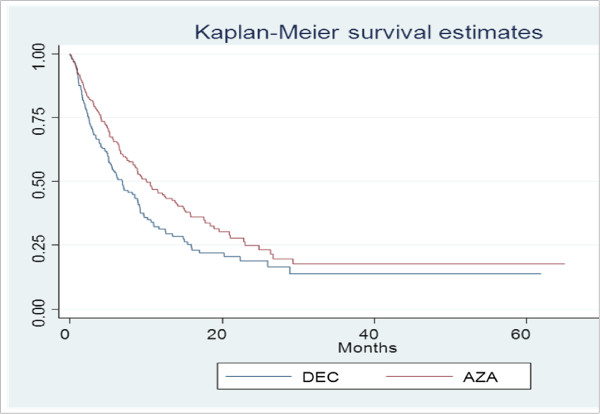
Overall survival.

Overall survival was significantly better in the AZA-treated cohort compared with patients in the DEC-treated cohort (10.1 months vs. 6.9 months respectively; p = 0.007, Figure 1) and treatment with azacitidine resulted in a significantly longer time to death when compared with decitabine treatment (adjusted HR 0.721, p = 0.008). Covariates (listed in Table 2) which were significantly associated with poorer survival were being male (adjusted HR 1.522, p = 0.001) and those who had a prior red blood cell (RBC) transfusion (adjusted HR 1.373, p = 0.018). Other covariates that were not independently associated with time to death include prior diagnosis of myelodysplastic syndrome (MDS) and type of insurance. After controlling for these variables, neither Charlson comorbidity score nor prior hospitalization were associated with time to death (Table 2).

**Table 2:** Time-to-event analyses: Cox PH models of mortality and hospitalization

	**Death**		**Hospitalization**	
	**Adjusted hazard ratio**	**p-value**	**Adjusted hazard ratio**	**p-value**
Azacitidine^a^	0.721	(0.008)**	0.787	(0.020)*
Age	1.031	(0.000)**	0.999	(0.818)
Gender Male	1.522	(0.001)**	1.097	(0.372)
Prior Diagnosis of MDS^b^	0.878	(0.283)	1.131	(0.225)
Charlson comorbidity index score^c^	0.937	(0.088)	1.001	(0.974)
Prior RBC transfusion^d^	1.373	(0.018)*	1.321	(0.012)*
Medicare Advantage enrollee^e^	0.973	(0.855)	0.993	(0.954)
Prior Hospitalization^f^	1.303	(0.069)	1.061	(0.618)
Observations	487		487	

p-values in parentheses.

*significant at 5%; ** significant at 1%.

Reference groups: ^a^decitabine, ^b^no diagnosis of MDS in the 6 months prior to starting treatment with a demethylating agent, ^c^Charlson comorbidity index is a continuous score from 0 to 24 (most comorbidity burden), ^d^no RBC transfusion in 6 months prior to starting treatment with a demethylating agent, ^e^commerical insurance, ^f^no hospitalizations in 6 months prior to starting treatment with a demethylating agent.

### Hospitalization

**Figure 2:**
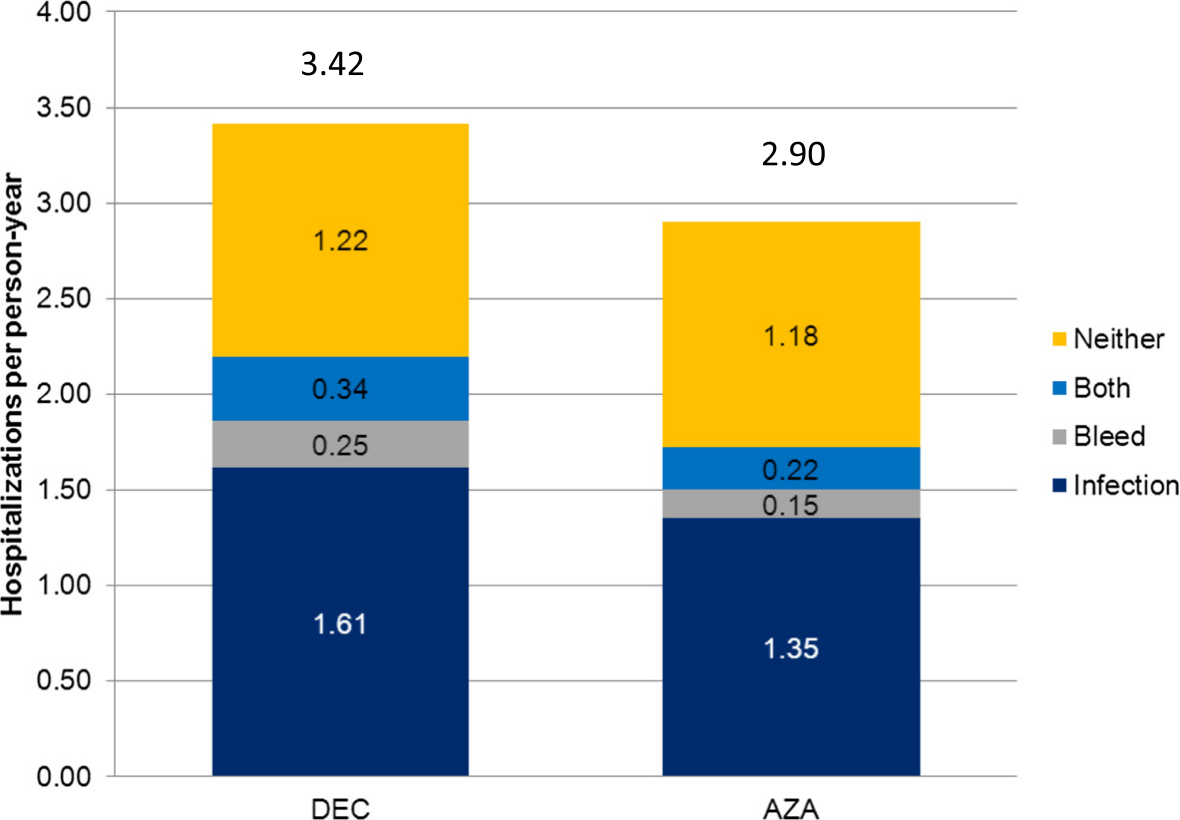
Reasons for hospitalization.

Overall hospitalization rates were lower in the AZA-treated cohort compared with the DEC-treated cohort (2.90 vs. 3.42 per person-year, Figure 2). Likewise, patients in the AZA-treated cohort also had a significantly longer median time to first hospitalization when compared with DEC-treated patients (1.9 vs. 1.4 months respectively; p = 0.015) and an overall lower risk of hospitalization (adjusted HR 0.787, p = 0.02) (Table 2). Prior RBC transfusions were found to significantly increase the time to hospitalization (adjusted HR 1.373, p = 0.018) while no other covariates examined were found to impact the risk of hospitalization. The primary reason for hospitalization in both cohorts were infections (AZA 46.6%, DEC 47.1%). Less common reasons for hospitalization are bleeding events (5.1% AZA, 7.3% DEC) and both a bleeding and infection event (7.6% AZA, 9.9% DEC). Just over one third of the hospitalizations in each cohort were not associated with either bleeding or infections.

## Discussion

This real-world study was designed to provide data to supplement clinical trial data and to answer the important question of the effectiveness of treatment in actual clinical practice. These findings are consistent with clinical trials in which the OS for azacitidine [9,15,16] has been found to be longer than the OS observed in trials of decitabine [11,12]. While, the median OS for AML patients treated with azacitidine in this study was shorter than what was reported in clinical trials [9,15,16], it was still longer than OS for patients treated with decitabine either in this retrospective study or previous trials [11,12]. Our findings were also consistent with the data generated in another usual care setting, which showed a similar magnitude of overall survival as the present analysis. Maurillo et al. reported that the OS for azacitidine in a non-trial context was a median of 9 months among patients who were newly-treated for AML (n = 35, median age 77) [17].

Real-world data regarding the effect of treatment on hospitalizations, bleeding and infections are very limited. Fenaux et al. found a similar hospitalization rate for azacitidine-treated patients (3.4 per patient year) [9]. Reported rates of infection (0.64 per patient-year) and bleeding (0.56 per patient year) rates for azacitidine-treated were lower than the rates found in this study [15]. These differences may be due to different methodological definitions of these events. Clearly, additional real-world evidence is needed to assess treatment associated hospitalization and comparative safety for therapeutic options available to treat AML.

Several limitations exist for retrospective claims analyses. Administrative claims data in general are limited by the potential for misclassification of diagnosis, study covariates, and/or outcomes. Most importantly, patients were not randomly assigned to treatment. Since common clinical measures of disease severity and patient prognosis (absolute neutrophil count [ANC] levels, platelet counts, and blast counts) are not available claims data, the two cohorts may be unbalanced with one cohort at higher risk for disease progression. This would be the case if physician select hypomethylating agents based on severity and prognosis. In our analyses we adjust for variables available in the claims data that are correlated with these measures (prior hospitalizations, MDS diagnoses, and red blood cell transfusions, use of erythropoietin-stimulating agents [ESA] and granulocyte colony-stimulating factors). Furthermore, we can find no published literature that suggests, or recommends, physicians select patients with a poor prognosis for treatment with either agent. Administration schedules for each therapy were not controlled and therefore dosing and/or administration variances may limit the results. While we recognize that dosing and adherence plays an important role in outcomes, this is a real-word study and the intent was to analyze how selection of treatment was related to outcomes. Death data likely underestimate total deaths, but we do not expect differential bias between cohorts.

## Conclusion

The clinical results determined by well-designed and controlled clinical trials are often not able to be duplicated when applied as standard of care outside of the pre-specified treatment, monitoring and support plans outlined by the clinical protocol. It is important to recognize this when assessing these “real-world” outcomes that show an overall survival advantage for AML patients who were treated with azacitidine compared to decitabine. The survival time in azacitidine-treated patients was further differentiated from those treated with decitabine by being associated with less time in the hospital. These data held up well after testing for numerous factors that may otherwise independently affect results. Although there are no studies that compare outcomes between patients randomly assigned to azacitidine or decitabine, physicians who choose to treat their AML patients with azacitidine outside of the confined of a clinical trial should have confidence in their choice.

## Methods

### Study design

This study was a retrospective, administrative claims-based analysis of patients diagnosed with acute myeloid leukemia who were treated with azacitidine or decitabine to assess real-world patient outcomes. Medical and pharmacy claims data used in the analysis were for services or products provided from January 1, 2006 through June 20, 2012.

### Data source

Data for this retrospective claims data-based analysis of patients from a large US commercial health plan were obtained from the Optum Research Database. The database contains de-identified medical and pharmacy claims data for over 33 million commercially-insured members annually, as well as eligibility information and linked mortality data from Social Security Administration death master files. Approximately 3.6 million Medicare Part C enrollees (commonly referred to as Medicare Advantage) and 5 million Medicare Part D enrollees since 2006 are included in the database. The population contained within the Optum Research Database is geographically diverse across the US, with a concentration of patients in the South, and therefor fairly representative of the U.S. population.

No identifiable protected health information was extracted or accessed during the course of the study. Pursuant to the Health Insurance Portability and Accountability Act, the use of de-identified data does not require Institutional Review Board approval or waiver of authorization.

### Study population

Subjects included were commercial or Medicare Advantage health plan members, ≥18 years of age with a diagnosis of AML (ICD-9-CM claim of 205.0x) in baseline or within 6 months prior to or within 60 days post initiation of treatment with either azacitidine (CPT J9025) or decitabine (CPT J0894) between January 1, 2006 and April 30, 2012 (index date). Continuous enrollment was required from 6 months prior to the index date (baseline period) to the earlier of death, disenrollment from the plan, or June 30, 2012. The follow-up period was variable and continued from the index date to the earlier of death, disenrollment from health plan, or June 30, 2012. Patients were excluded if they were <18 years of age at index date or had a claim for azacitidine or decitabine in the baseline period. Two cohorts were created based on treatment (azacitidine-treated patients and decitabine-treated patients).

### Demographic and clinical characteristics

Patient demographics examined at index for each treatment include age, gender, geographic region (Northeast, Midwest, South, West) and insurance type (commercial or Medicare Advantage). Clinical characteristics examined include treatment type (azacitidine or decitabine), history of MDS diagnosis and RBC transfusion in baseline period and baseline or post-index ESA and granulocyte or granulocyte-macrophage colony-stimulating factor use. Baseline calculations were performed to identify the Quan Charlson comorbidity score [18] and hospitalization.

### Patient outcomes

The primary patient outcome was OS, calculated as the time period from the index date until the date of death. A second outcome examined was hospitalization. All outcomes were measured in the post-index period, inclusive of the index date. The overall number of hospitalizations was measured and calculated as ‘hospitalizations per person-year’. Reasons for hospitalization were also captured based on the primary diagnosis code listed on the claim. These specific hospitalizations include infection, bleed, and cardiac related events. Finally, the time to first hospitalization after the index date was calculated for both all-cause and AML-related hospitalizations.

Use of a transfusion was determined based on evidence of a claim for at least one red blood transfusion during the follow-up period. Transfusion dependence was defined as those with evidence (a claim in any position on claim form) of at least two transfusion events on separate days during an eight-week period as indicated by procedure and/or revenue codes and service dates for whole or red blood cell transfusions. The service date for the first transfusion in the first episode of transfusion-dependence in the follow-up period is defined as the transfusion dependence.

### Statistical analysis

Unadjusted comparisons of OS and time to first hospitalization between azacitidine and decitabine patients were made via Kaplan-Meier estimator to account for variable length of follow-up. A Cox proportional hazards model was used to examine the relationship between choice of demethylating agent and OS, time to 1st hospitalization, and transfusion dependence while controlling for age, gender, comorbidity score, prior MDS diagnosis, prior red blood cell transfusion, prior hospitalizations, and insurance type. All analyses were conducted using version 10.1 of the STATA/SE software package (Stata Corp, College Station, TX).

## Competing interests

The study and manuscript preparation were funded by Celgene. B. Douglas Smith, MD declares that he has no competing interests. Charles L. Beach, PharmD, Dalia Mahmoud, MBA, and Laura Weber are employees of Celgene and hold equity. Henry J. Henk, PhD is an employee of Optum, which was contracted by Celgene to conduct the study.

## Authors’ contributions

All authors participated in the design of the study. HJH designed and conducted the retrospective claims research and performed the statistical analysis. All authors participated in drafting the manuscript. All authors read and approved the final manuscript.
