# Correction: Survival and hospitalization among patients with acute myeloid leukemia treated with azacitidine or decitabine in a large managed care population: a real-world, retrospective, claims-based, comparative analysis

**DOI:** 10.1186/2162-3619-3-19

**Published:** 2014-07-16

**Authors:** B Douglas Smith, Charles L Beach, Dalia Mahmoud, Laura Weber, Henry J Henk

**Affiliations:** 1 Sidney Kimmel Comprehensive Cancer Center at Johns Hopkins, Baltimore, MD, USA; 2 Hematology/Oncology Clinical Research and Development, Celgene, Summit, NJ, USA; 3 Global Pricing and Market Access, Celgene, Summit, NJ, USA; 4 Health Economic and Outcomes Research, Optum, Eden Prairie, MN, USA

## 

After the publication of this work [1], it was brought to our attention that a statement in the article is not consistent with the data. The statement “Prior RBC transfusions were found to significantly increase the time to hospitalization (adjusted HR 1.373, p = 0.018) while no other covariates examined were found to impact the risk of hospitalization” is not a correct reflection of the results from the data analysis in Table 2 of the article.

The corrected statement is provided here as follows:

“Prior RBC transfusions were found to significantly shorten the time to hospitalization (adjusted HR 1.373, p = 0.018) while no other covariates examined were found to impact the risk of hospitalization.”
